# Comparison of efficacy of exosomes derived from human umbilical cord blood mesenchymal stem cells in treating mouse acute lung injury via different routes

**DOI:** 10.3389/fped.2025.1560915

**Published:** 2025-05-29

**Authors:** Jing Chen, Shuang Liu, Jizhen Zou, Yi Wang, Haiyan Ge, Yi Hui, Siyuan Huang, Wei Li, Weilan Na, Xiaolan Huang, Lin Bai, Yiying Huang, Dong Qu

**Affiliations:** ^1^ Department of Critical Medicine, Children’s Hospital Affiliated Capital Institute of Pediatrics, Beijing, China; ^2^ Department of Critical Care Medicine, Capital Institute of Pediatrics, Beijing, China; ^3^ Beijing Municipal Key Laboratory of Child Development and Nutriomics, Capital Institute of Pediatrics, Beijing, China; ^4^ Experimental Research Center, Capital Institute of Pediatrics, Beijing, China; ^5^ Institute of Laboratory Animal Science, (CAMS & PUMC), Beijing, China

**Keywords:** acute lung injury, acute respiratory distress syndrome, mesenchymal stem cells, exosomes, cytokines

## Abstract

**Objective:**

To investigate the therapeutic efficacy of human umbilical cord blood mesenchymal stem cell-derived exosomes (hUCMSC-Exo) in a lipopolysaccharide (LPS)-induced acute lung injury (ALI) mouse model and compare the effects of different administration routes.

**Methods:**

An ALI mouse model was established through intratracheal LPS injection. Mice received hUCMSC-Exo through tail vein injection, nasal drip, or atomization at 4-and-24 h post-modeling, with comparisons made across low, medium, and high doses. Mice were categorized into three groups: control, LPS model, and experimental (*n* = 8). Histopathological scoring assessed lung inflammation after 48 h; and inflammatory cytokine levels (TNF-α, IL-6, IL-1β, and IL-10) in serum and bronchoalveolar lavage fluid (BALF) were quantified by enzyme-linked immunosorbent assay (ELISA).

**Results:**

In a murine model of LPS-induced ALI, administration of hUCMSC-Exo via intravenous, intranasal, or nebulized routes at 4 and 24 h post-LPS exposure significantly attenuated pulmonary inflammation, as evidenced by reduced alveolar inflammatory cell infiltration, hemorrhage, and edema in histopathological analysis (except the nebulized low-dose group). ELISA revealed that hUCMSC-Exo markedly decreased serum and bronchoalveolar lavage fluid (BALF) levels of pro-inflammatory cytokines TNF-α, IL-6, and IL-1β (*P* < 0.05) while increasing IL-10 levels. Dose-dependent effects were observed across routes: intravenous high-dose (Exo-VH) outperformed medium- and low-dose groups (*P* < 0.05); intranasal medium-dose (Exo-NM) was superior to low-dose (Exo-NL; *P* < 0.05), with no significant difference between medium and high doses (*P* > 0.05); nebulized high-dose (Exo-AH) demonstrated enhanced efficacy over medium- (Exo-AM; *P* < 0.05) and low-dose (Exo-AL; *P* < 0.05). At an equivalent dose (5 × 10⁸ particles), intravenous delivery achieved superior lung injury score reduction and cytokine modulation compared to intranasal and nebulized routes (*P* < 0.05), whereas the latter two showed comparable efficacy (*P* > 0.05). These findings collectively highlight the therapeutic potential of hUCMSC-Exo in ALI, with intravenous administration emerging as the optimal route at the tested dose.

**Conclusion:**

hUCMSC-Exo effectively attenuates LPS-induced ALI in mice. At the tested dose (5 × 10⁸ particles), intravenous delivery exhibited superior therapeutic efficacy over intranasal and nebulized routes.

## 1 Introduction

Acute lung injury (ALI) and its severe form, acute respiratory distress syndrome (ARDS), are associated with high morbidity and mortality in clinical practice, with mortality rates reaching 35%–50% among critically ill ARDS patients in intensive care units (ICUs) (1). Current clinical management of ALI/ARDS lacks curative therapies and primarily relies on supportive measures such as mechanical ventilation and fluid management, underscoring the urgent need to explore novel therapeutic strategies.

The pathogenesis of ARDS is multifaceted, with core mechanisms involving damage to the pulmonary vascular endothelium and alveolar epithelium (2). Underlying causes include direct bacterial or viral invasion, excessive immune activation, and mechanical ventilation-induced stretch injury. These insults trigger massive infiltration of inflammatory cells (e.g., neutrophils, macrophages) into lung tissues, releasing pro-inflammatory cytokines such as tumor necrosis factor-α (TNF-α) and interleukin-1β (IL-1β). These cytokines activate nuclear factor-kappa B (NF-κB) signaling pathways, amplifying pulmonary edema and inflammatory cascades (3, 4), disrupting the alveolar-capillary barrier, increasing vascular permeability, and promoting protein-rich fluid leakage into alveoli. This results in impaired gas exchange and eventual respiratory failure.

Recently, cell-based therapies have emerged as promising interventions for ARDS, with mesenchymal stem cell-derived exosomes (MSC-Exo) at the forefront (5–7). Exosomes are 30–150 nm nanovesicles secreted by cells, carrying bioactive molecules including proteins, nucleic acids (mRNA, miRNA), and lipids (8). By delivering miRNAs, exosomes regulate multiple signaling pathways (e.g., NF-κB, Wnt/*β*-catenin) to modulate target cell functions (9). Compared to traditional cell therapies, exosomes offer advantages in stability, efficacy, non-immunogenicity, and lack of microvascular occlusion risk, with proven safety (10–13). Preclinical studies demonstrate that exosome therapy improves pulmonary inflammation, optimizes lung architecture, enhances vascular remodeling, restores exercise capacity, and ameliorates ALI/ARDS outcomes (6, 14–16). Various administration routes (intratracheal, intraperitoneal, intravenous, nebulization) show therapeutic benefits across different ALI models (17–22), supporting the rationale for cytokine-targeted immune modulation.

Despite promising results in adult models, exosome-based therapies for pediatric pulmonary diseases remain in their infancy. Clinical translation faces multiple barriers: (1) Immune system immaturity in children may lead to distinct exosome mechanisms compared to adults (23); (2) Ethical constraints and limited accessibility of pediatric samples hinder research progress; (3) Standardized dosing protocols, frequency, and treatment duration for pediatric exosome therapy remain undefined (16). Existing preclinical studies lack systematic comparisons of safety and efficacy across administration routes (intratracheal, intranasal, intraperitoneal, intravenous). Future research must elucidate the developmental biology of exosomes in pediatric lungs and establish personalized, developmental stage-tailored therapeutic regimens to advance precision medicine for pediatric pulmonary diseases.

To address these challenges, this study establishes an LPS-induced ALI model in mice to evaluate the therapeutic efficacy of exosomes in murine ALI and identify the optimal administration route. By analyzing histopathological changes in lung tissue and cytokine concentrations (TNF-α, IL-6, IL-1β, IL-10) in serum and bronchoalveolar lavage fluid (BALF) at 48 h post-treatment, we compare the restorative effects of intravenous injection, intranasal instillation, and nebulization. Findings aim to provide mechanistic insights and guide clinical translation for exosome-based ALI therapies.

## 2 Materials and methods

### 2.1 Animals

Female BALB/c mice (6–8 weeks old, 19–21 g) were purchased from Beijing Vital River Laboratory Animal Technology Co., Ltd. [license number: SCXK (Jing) 2021-0011]. Mice were maintained at the Institute of Laboratory Animal Science, Chinese Academy of Medical Sciences, under controlled conditions (temperature: 25°C, humidity: 50%–60%) with *ad libitum* access to food and water. All animal procedures complied with the Guide for the Care and Use of Laboratory Animals and were approved by the Institutional Animal Care and Use Committee (IACUC) of the Chinese Academy of Medical Sciences (approval number: BL21003).

### 2.2 LPS-Induced ALI model

The ALI model was established as previously described (24). Briefly, mice were anesthetized via intraperitoneal injection of pentobarbital sodium (40 mg/kg). A single intratracheal dose of 50 μl sterile saline containing 100 μg LPS (Sigma-Aldrich, catalog no. L2630; strain 0111:B4) was administered. Mice were recovered in a 100% oxygen chamber and subsequently euthanized. Lung tissues were collected for histopathological analysis, including hematoxylin and eosin (H&E) staining to assess inflammatory cell infiltration, alveolar edema, hemorrhage, interstitial thickening, and hyaline membrane formation.

### 2.3 hUCMSC-Exo administration

**Figure 1:**
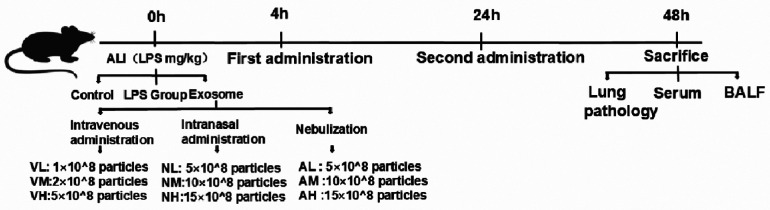
Administration protocol of hUCMSC-Exo in a murine model of LPS-induced ALI.

Following LPS-induced ALI, mice were randomized into control (*n* = 4), LPS model (*n* = 4), and experimental groups (*n* = 8) using a randomization table. Experimental groups were further divided into nine subgroups (*n* = 8 per subgroup) receiving intravenous, intranasal, or nebulized delivery of hUCMSC-Exo (Shandong Umbilical Cord Blood Stem Cell Bank) at 4 and 24 h post-LPS exposure. Doses were optimized based on published ranges (10⁶–10¹⁰ particles/mouse), with medium-dose exosomes (10 × 10⁸ particles/mouse) prioritized for primary analysis. Subgroup-specific doses were as follows: i.v. low (Exo-VL, 1 × 10⁸), medium (Exo-VM, 2 × 10⁸), and high (Exo-VH, 5 × 10⁸); i.n. low (Exo-NL, 5 × 10⁸), medium (Exo-NM, 10 × 10⁸), and high (Exo-NH, 15 × 10⁸); nebulized low (Exo-AL, 5 × 10⁸), medium (Exo-AM, 10 × 10⁸), and high (Exo-AH,15 × 10⁸). All protocols adhered to standardized exosome preparation and administration procedures, with detailed methodologies illustrated in Figure 1.

### 2.4 Lung injury scoring

At 48 h post-exosome treatment, the left lung tissues from each mouse were collected, fixed in 4% paraformaldehyde, paraffin-embedded, and sectioned into 4 μm-thick slices. Paraffin sections underwent deparaffinization with xylene (I and II, 10–15 min each), followed by gradient ethanol dehydration (100% → 70%). Hematoxylin and eosin (H&E) staining was performed as per Histological Techniques (3rd edition): hematoxylin staining for 3–8 min, 1% hydrochloric acid-ethanol differentiation for 30 s to 1 min, running water bluing for 5–10 min, eosin staining for 1–3 min, and dehydration/transparentizing with gradient ethanol (70% → 100%) prior to mounting. Frozen sections were processed via 10–30 s fixation, hematoxylin staining at 60°C for 30–60 s, bluing for 5–10 s, eosin staining for 30–60 s, and dehydration/transparentizing. Pathological damage was evaluated using the “Smith Lung Injury Scoring” criteria (25) (Table 1). Histopathological changes, including inflammatory cell infiltration, alveolar congestion/hemorrhage, edema, and interstitial thickening/hyaline membrane formation, were scored on a 0–4 scale under light microscopy: 0 = no injury; 1 = mild injury (≤25% affected area); 2 = moderate injury (26%–50%); 3 = severe injury (51%–75%); 4 = critical injury (>75%). Total scores were calculated by summing individual category scores.

**Table 1:** Smith pulmonary histopathology injury scoring criteria.

Parameter	0	1	2	3	4
Alveolar Edema	None	<25% range	25%–50% range	51%–75% range	>75% range
Alveolar/Interstitial Inflammation	None	<25% range	25%–50% range	51%–75% range	>75% range
Alveolar/Interstitial Hemorrhage	None	<25% range	25%–50% range	51%–75% range	>75% range
Atelectasis/Hyaline Membrane Formation	None	<25% range	25%–50% range	51%–75% range	>75% range

### 2.5 Cytokine analysis

Mice were euthanized via cervical dislocation at 48 h post-treatment. BALF was collected by instilling 1 ml phosphate-buffered saline (PBS) into the lungs via a 2.5 ml syringe, followed by three sequential lavages. BALF and blood samples (1 ml, collected via orbital puncture) were centrifuged at 3,000 × g for 10 min at 4°C. Supernatants were stored at −80°C. Cytokine levels (IL-6, IL-10, IL-1β, TNF-α) in serum and BALF were quantified using ELISA kits (Shanghai Hapex Biotech Co., Ltd.) following the manufacturer's protocol. Briefly, samples were added to 96-well ELISA plates, incubated at room temperature for 2 h, and detected at 450 nm.

### 2.6 Statistical analysis

Data were analyzed using GraphPad Prism 8.0 (GraphPad Software, San Diego, CA, USA). Normally distributed data are expressed as mean ± standard deviation (mean ± SD). Intergroup comparisons were performed using Student's t-test, while multiple group comparisons were conducted via one-way analysis of variance (ANOVA) followed by Tukey's *post hoc* test. Statistical significance was defined as *P* < 0.05.

## 3 Results

### 3.1 Impact of exosome administration routes and doses on pulmonary injury scores in LPS-induced ALI mice

**Figure 2:**
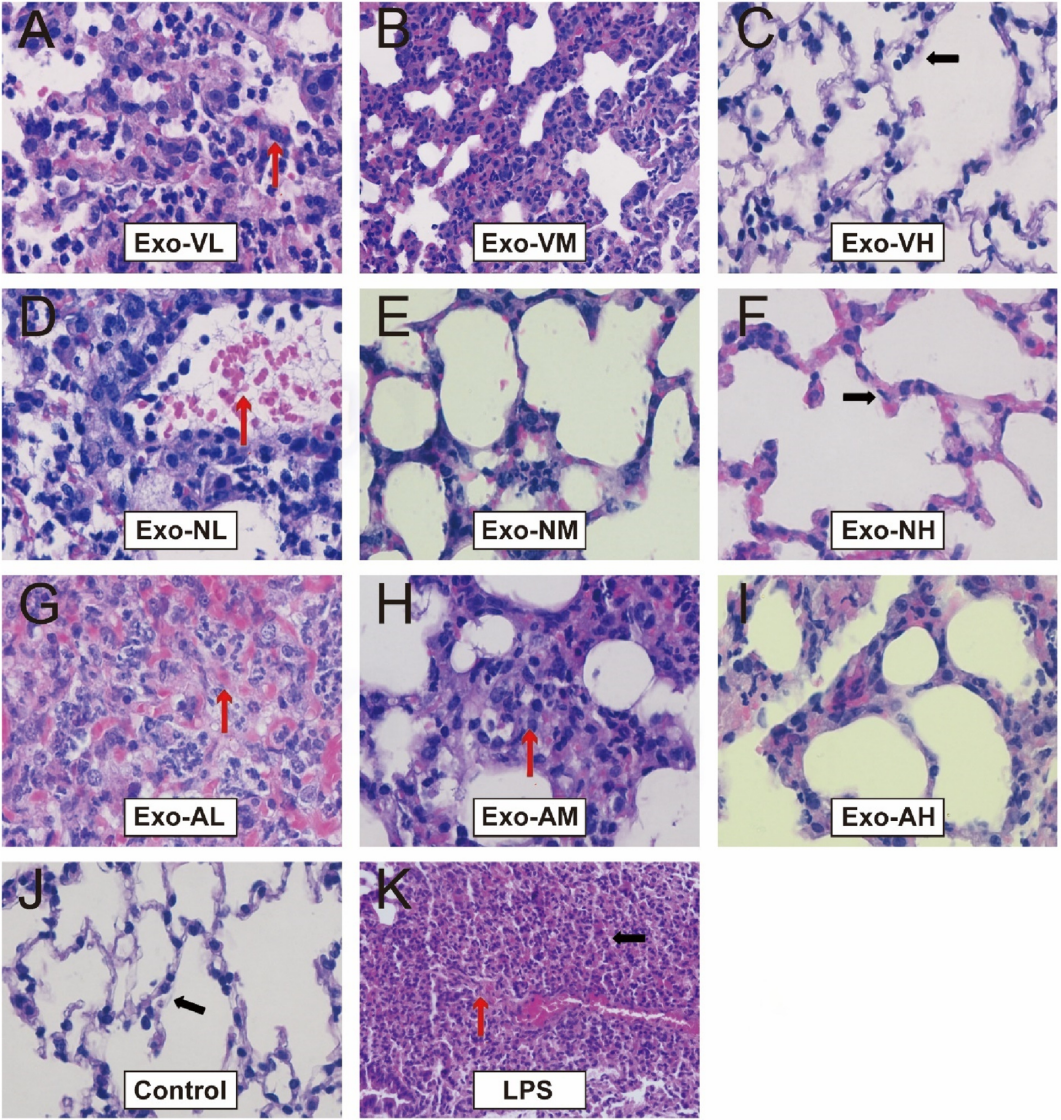
H&E staining of lung tissues at 48 hours post-LPS-induced ALI. Representative histopathological images of lung tissues from control, LPS model, and exosome-treated groups (intravenous, intranasal, nebulized; low-, medium-, high-dose) at ×400 magnification (*n* = 4; images captured from three random fields per tissue section). LPS model group exhibited significant alveolar structural disruption (black arrows), including alveolar wall rupture, inflammatory cell infiltration, and interstitial edema. Exosome-treated groups demonstrated marked attenuation of lung injury, with preserved alveolar architecture (red arrows indicating intact alveolar septa) and reduced congestion and interstitial thickening.

**Figure 3:**
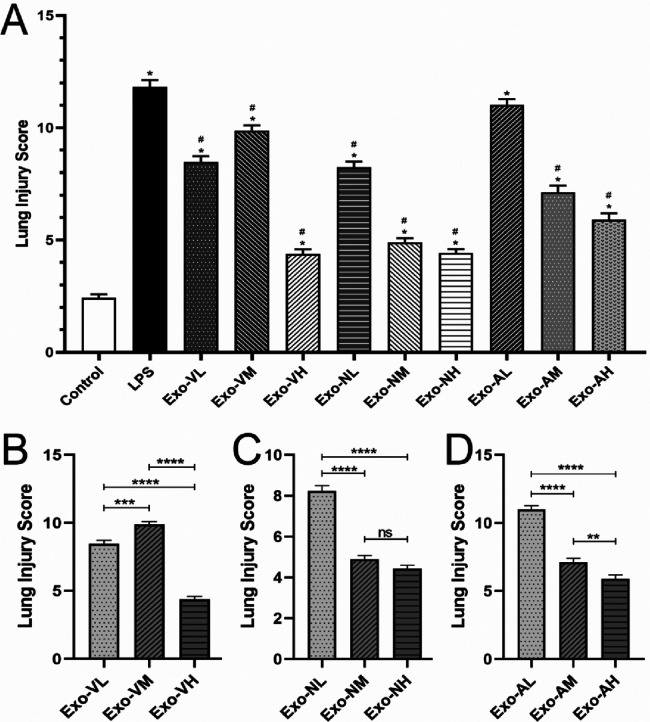
Lung injury scores in LPS-induced ALI mice at 48 hours post-treatment. All data were derived from the same cohort of mice, with experimental procedures outlined in Figure 1. Five random fields per lung section (*n* = 4/group) were analyzed. **(A)** Exosome-treated groups (intravenous, intranasal, nebulized) showed significant reductions in total injury scores compared to the LPS model group (*P* < 0.05), with the intravenous high-dose group (Exo-VH) demonstrating the most pronounced improvement. **(B)** Dose-dependent effects were observed for intravenous delivery: high-dose exosomes (Exo-VH) outperformed medium- (Exo-VM) and low-dose (Exo-VL) groups (*P* < 0.05). **(C)** For intranasal administration, medium-dose exosomes (Exo-NM) significantly reduced injury scores vs. low-dose (Exo-NL; *P* < 0.05), while medium- and high-dose (Exo-NH) groups did not differ (*P* = 0.2326). **(D)** Nebulized delivery exhibited a stepwise dose response, with high-dose (Exo-AH) > medium-dose (Exo-AM; *P* < 0.05) > low-dose (Exo-NL; *P* < 0.05). Statistical significance is denoted as *P* < 0.05 vs. control (#) or LPS model (*).

At 48 h post-LPS exposure, histopathological analysis revealed characteristic ALI features, including alveolar wall thickening, inflammatory cell infiltration, and interstitial expansion (Figure 2K). Exosome treatment significantly mitigated lung injury across all routes, with dose-dependent reductions in inflammatory infiltration, hemorrhage, and edema compared to the LPS model group (Figures 2A–I, 3A). Among the three routes, intravenous delivery demonstrated the most pronounced dose effect: high-dose exosomes (Exo-VH) significantly outperformed medium- (Exo-VM) and low-dose (Exo-VL) groups (*P* < 0.05; Figure 3B). Intranasal administration showed moderate dose dependency, where medium-dose (Exo-NM) outperformed low-dose (Exo-NL; *P* < 0.05), while medium- and high-dose (Exo-NH) groups did not differ significantly (*P* = 0.2326; Figure 3C). Nebulized delivery exhibited a stepwise improvement, with high-dose (Exo-AH) superior to medium- (Exo-AM; *P* < 0.05) and low-dose (Exo-AL; *P* < 0.05) groups (Figure 3D). These findings collectively highlight the therapeutic potential of exosomes in ALI, with intravenous high-dose and nebulized medium/high-dose regimens showing the greatest efficacy. The lung Injury scores across treatment groups are further explored in Table 2.

**Table 2:** Comparative analysis of lung injury scores and statistical significance across groups.

Group	Pathology Score (Mean ± SD, *n* = 4)	*P*-value
Healthy	2.429 ± 1.336	
LPS	11.81 ± 2.130*	
Exo-VL	8.475 ± 2.317*^,#^	<0.0001
Exo-VM	9.875 ± 2.052*^,#^	<0.0001
Exo-VH	4.375 ± 1.925*^,#^	<0.0001
Exo-NL	8.238 ± 2.291*^,#^	<0.0001
Exo-NM	4.900 ± 1.604*^,#^	<0.0001
Exo-NH	4.425 ± 1.516*^,#^	<0.0001
Exo-AL	11.010 ± 2.319*	<0.0001
Exo-AM	7.125 ± 2.650*^,#^	0.1952
Exo-AH	5.900 ± 2.578*^,#^	<0.0001

Results are expressed as mean ± standard deviation ($\bar{X}\pm s$, *N* = 4).

Statistical significance is denoted as *P < 0.05 vs. control group; ^#^*P* < 0.05 vs. LPS model group.

### 3.2 Exosome-mediated modulation of serum cytokines in LPS-induced ALI mice

**Figure 4:**
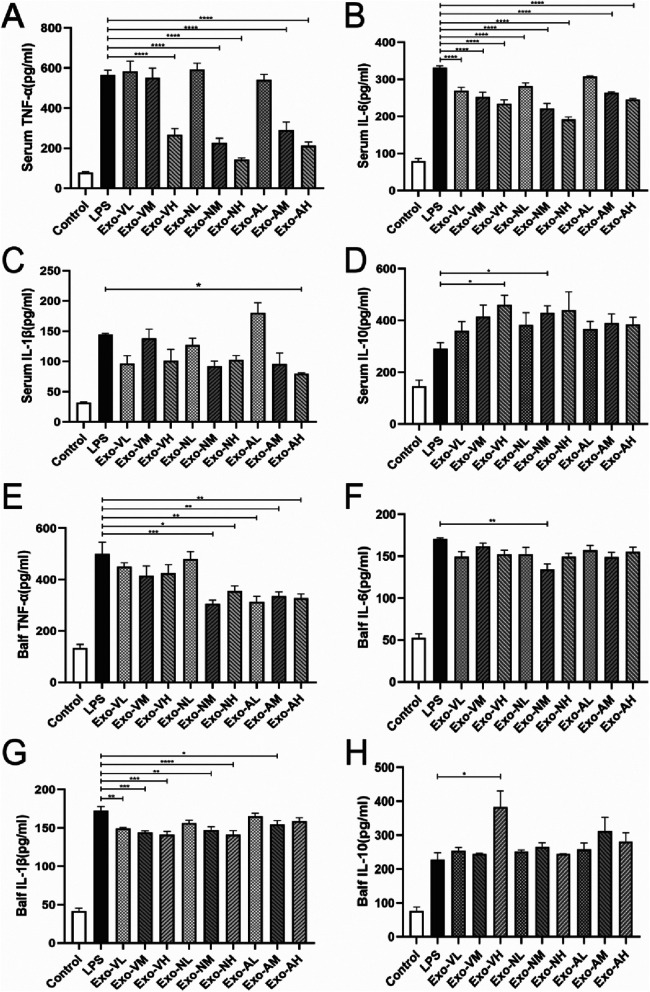
Exosome-Mediated modulation of inflammatory cytokine profiles in Serum and bronchoalveolar lavage fluid (BALF) of LPS-induced ALI mice. Exosome treatment differentially regulated pro-inflammatory and anti-inflammatory cytokine levels in serum and BALF of LPS-challenged mice at 48 h post-administration. **(A)** Serum TNF-α, **(B)** serum IL-6, **(C)** serum IL-1β, **(D)** serum IL-10, **(E)** BALF TNF-α, **(F)** BALF IL-6, **(G)** BALF IL-1β, and **(H)** BALF IL-10 concentrations in each treatment group. *P* < 0.05, *P* < 0.005, *P* < 0.0001, ****P < 0.0001 vs. LPS model group.

Elevated levels of TNF-α, IL-6, and IL-1β indicate pro-inflammatory responses, whereas IL-10, with its anti-inflammatory and immunosuppressive roles, suppresses pro-inflammatory cytokine production, inhibits inflammatory cell activity, modulates T-cell function, and restrains B-cell activation and antibody secretion, thereby reflecting anti-inflammatory regulation (26). At 48 h post-exosome administration, serum TNF-α and IL-6 levels were significantly reduced compared to the LPS model group, while IL-10 exhibited an upward trend. Exosome-treated groups demonstrated a consistent pattern of reduced TNF-α/IL-1β and elevated IL-10 (Figures 4A–D).

#### 3.2.1 TNF-α modulation by exosome therapy in LPS-induced ALI mice

Serum TNF-α concentrations in all exosome-treated groups were significantly elevated compared to the control group [(78.1 ± 9.5) pg/ml], but markedly reduced vs. the LPS model group [(565.0 ± 49.3) pg/ml], demonstrating dose- and route-dependent therapeutic effects. Specifically, high-dose intravenous exosomes (Exo-VH; 266.8 ± 29.2 pg/ml) outperformed medium- (Exo-VM; 266.8 ± 29.2 pg/ml) and low-dose (Exo-VL; 266.8 ± 29.2 pg/ml) groups (*P* = 0.0015 and *P* = 0.0029, respectively), with no significant difference between medium- and low-dose (*P* = 0.8780). For intranasal delivery, medium- (Exo-NM; 226.6 ± 57.3 pg/ml) and high-dose (Exo-NH; 142.5 ± 22.0 pg/ml) exosomes significantly lowered TNF-α vs. low-dose (Exo-NL; *P* < 0.0001 for both), while medium- and high-dose groups did not differ (*P* = 0.0572). Nebulized administration showed similar trends, with high- (Exo-AH; 211.6 ± 39.4 pg/ml) and medium-dose (Exo-AM; 289.4 ± 73.0 pg/ml) exosomes significantly reducing TNF-α compared to low-dose (Exo-AL; *P* = 0.0018 and *P* = 0.0002, respectively), though medium-and high-dose efficacy did not differ (*P* = 0.2073). The serum TNF-α concentrations across treatment groups are further explored in Table 3.

**Table 3:** TNF-α concentrations across treatment groups.

Group	$\mathrm{TNF}\text{-}\alpha \;(\bar{X}\pm s)$	*P*-value
Control	78.1 ± 9.4^#^	
LPS	565.0 ± 49.3*	
Exo-VL	582.7 ± 90.7*	>0.9999
Exo-VM	551.1 ± 84.3*	>0.9999
Exo-VH	266.8 ± 69.2*^,^^#^	<0.0001
Exo-NL	592.9 ± 70.5*	0.9896
Exo-NM	226.6 ± 57.2*^,^^#^	<0.0001
Exo-NH	142.5 ± 22.0^#^	<0.0001
Exo-AL	540.4 ± 48.5*	0.9985
Exo-AM	289.4 ± 73.0*^,^^#^	<0.0001
Exo-AH	211.6 ± 39.4*^,^^#^	<0.0001

Values expressed as mean ± SD $\left(\bar{X}\pm s\right)$ with sample size (*N* = 4) in parentheses <0.05 vs. control.

*Statistical significance compared to control group.

^#^ *P* < 0.05 vs. LPS model.

#### 3.2.2 IL-6 modulation by exosome therapy in LPS-induced ALI mice

Exosome treatment significantly reduced serum IL-6 levels compared to the LPS model group [(331.7 ± 11.5) pg/ml], with most groups achieving statistical significance (*P* < 0.05), except the nebulized low-dose group (Exo-AL: 307.9 ± 3.5 pg/ml). Among the effective doses, intravenous high-dose (Exo-VH: 234.2 ± 23.5 pg/ml), intranasal medium-dose (Exo-NM: 221.9 ± 26.2 pg/ml), and nebulized high-dose (Exo-AH: 245.3 ± 4.8 pg/ml) exosomes demonstrated the most pronounced reductions (*P* < 0.0001 vs. LPS model). Dose-dependent effects varied by route: intravenous delivery showed no significant differences across low-, medium-, or high-dose groups (*P* > 0.05), whereas intranasal administration exhibited progressive dose-dependent reductions, with high- and medium-dose exosomes outperforming low-dose groups (*P* = 0.0014 and *P* < 0.0001, respectively), though high- and medium-dose efficacy did not differ (*P* = 0.1243). Nebulized delivery showed a consistent dose-dependent decrease in IL-6 levels (*P* < 0.05 for trend). Notably, the nebulized low-dose group (Exo-AL) failed to significantly reduce IL-6 compared to the LPS model. The serum IL-6 concentrations across treatment groups are further explored in Table 4.

**Table 4:** IL-6 concentrations across treatment groups.

Group	$\mathrm{IL}\text{-}6\;(\bar{X}\pm s)$	*P-*value
Control	79.4 ± 13.7^#^	
LPS	331.7 ± 11.5*	
Exo-VL	268.1 ± 17.5*^,^^#^	<0.0001
Exo-VM	252.7 ± 21.7*^,^^#^	<0.0001
Exo-VH	234.2 ± 23.5*^,^^#^	<0.0001
Exo-NL	282.0 ± 23.6*^,^^#^	<0.0001
Exo-NM	221.9 ± 26.2*^,^^#^	<0.0001
Exo-NH	191.6 ± 17.6*^,^^#^	<0.0001
Exo-AL	307.9 ± 3.5*	0.2030
Exo-AM	263.6 ± 6.2*^,^^#^	<0.0001
Exo-AH	245.3 ± 4.8*^,^^#^	<0.0001

Values expressed as mean ± SD $\left(\bar{X}\pm s\right)$ with sample size (*N* = 4) in parentheses <0.05 vs. control.

*Statistical significance compared to control group.

^#^ *P* < 0.05 vs. LPS model.

#### 3.2.3 IL-1β modulation by exosome therapy in LPS-induced ALI mice

Exosome treatment modulated serum IL-1β levels in a dose- and route-dependent manner. All experimental groups exhibited elevated IL-1β compared to healthy controls [(32.2 ± 1.9) pg/ml], except the nebulized high-dose group (Exo-AH: 79.5 ± 2.9 pg/ml), which significantly reduced IL-1β vs. the LPS model group [(144.2 ± 4.3) pg/ml] (*P* = 0.0432). Other exosome-treated groups, including intravenous high-dose (Exo-VH: 128.6 ± 9.1 pg/ml), intranasal medium-dose (Exo-NM: 132.4 ± 8.7 pg/ml), and nebulized medium-dose (Exo-AM: 104.2 ± 6.5 pg/ml), showed no significant difference from the LPS model (*P* > 0.05). Dose-dependent effects were route-specific: intravenous and intranasal administrations displayed no significant intra-dose variations (*P* > 0.05), whereas nebulized delivery demonstrated progressive reductions, with high- (Exo-AH) and medium-dose (Exo-AM) exosomes outperforming low-dose (Exo-AL: 140.1 ± 5.8 pg/ml) groups (*P* = 0.0108 and *P* = 0.0077, respectively), though high- and medium-dose efficacy did not differ (*P* = 0.7891). The serum IL-1β concentrations across treatment groups are further explored in Table 5.

**Table 5:** IL-1β concentrations across treatment groups.

Group	$\mathrm{IL}\text{-}1\beta \;(\bar{X}\pm s)$	*P*-value
Control	32.19 ± 1.9^#^	
LPS	144.2 ± 4.3*	
Exo-VL	96.33 ± 37.0	0.1110
Exo-VM	138.1 ± 36.9*	>0.9999
Exo-VH	101.1 ± 46.3*	0.2252
Exo-NL	127.3 ± 29.3*	0.9578
Exo-NM	92.4 ± 18.8	0.1181
Exo-NH	102.5 ± 20.3*	0.2083
Exo-AL	123.4 ± 66.6*	0.4992
Exo-AM	95.45 ± 48.8	0.1139
Exo-AH	79.54 ± 2.9	0.0432

Values expressed as mean ± SD $\left(\bar{X}\pm s\right)$ with sample size (*N* = 4) in parentheses <0.05 vs. control.

*Statistical significance compared to control group.

^#^ *P* < 0.05 vs. LPS model.

#### 3.2.4 IL-10 modulation by exosome therapy in LPS-induced ALI mice

Exosome therapy significantly elevated serum IL-10 levels compared to the LPS model group [(291.5 ± 56.96) pg/ml], with all treatment groups exceeding baseline levels observed in healthy controls [(146.4 ± 40.0) pg/ml]. High-dose intravenous (Exo-VH: 460.8 ± 83.8 pg/ml) and medium-dose intranasal (Exo-NM: 430.0 ± 76.0 pg/ml) exosomes demonstrated superior IL-10 induction vs. LPS model (*P* = 0.0203* and *P* = 0.0404*, respectively), while other dosages showed no significant difference. Notably, no dose-dependent effects were observed within the same administration route (intravenous, intranasal, or nebulized), as IL-10 levels remained comparable across low-, medium-, and high-dose groups (*P* > 0.05). The serum IL-10 concentrations across treatment groups are further explored in Table 6.

**Table 6:** IL-10 concentrations across treatment groups.

Group	$\mathrm{IL}\text{-}10\;(\bar{X}\pm s)$	*P*-value
Control	146.4 ± 40.0	
LPS	291.5 ± 57.0	
Exo-VL	360.5 ± 94.9*	0.6859
Exo-VM	414.4 ± 112.7*	0.1286
Exo-VH	460.8 ± 83.8*^,^^#^	0.0203
Exo-NL	382.8 ± 115.5*	0.4109
Exo-NM	430.0 ± 76.0*^,^^#^	0.0404
Exo-NH	439.7 ± 124.0*	0.1394
Exo-AL	368.0 ± 64.0*	0.6672
Exo-AM	389.4 ± 94.3*	0.2935
Exo-AH	384.8 ± 72.2*	0.3444

Values expressed as mean ± SD $\left(\bar{X}\pm s\right)$ with sample size (*N* = 4) in parentheses <0.05 vs. control.

*Statistical significance compared to control group.

^#^ *P* < 0.05 vs. LPS model.

### 3.3 Effects of different exosome administration routes and doses on BALF cytokines in LPS-induced ALI mice

At 48 h post-administration, exosome-treated groups exhibited significant reductions in pro-inflammatory cytokines TNF-α and IL-1β, alongside elevated anti-inflammatory IL-10 levels in BALF compared to the LPS model group (Figures 4E–H). Intravenous high-dose exosomes (Exo-VH) demonstrated the most pronounced suppression of TNF-α (*P* < 0.01) and IL-1β (*P* < 0.05), while intranasal and nebulized medium-dose exosomes significantly upregulated IL-10 (*P* < 0.05 vs. LPS model). Dose-dependent effects were route-specific: intravenous delivery showed no significant differences between medium- and high-dose groups (*P* > 0.05), whereas nebulized administration exhibited progressive reductions in TNF-α and IL-1β with escalating doses (*P* < 0.05 for trend). These findings collectively underscore the therapeutic efficacy of exosome-mediated cytokine modulation in ALI, with route and dose-optimized strategies critical for targeting inflammatory pathways.

#### 3.3.1 TNF-αModulation in BALF following exosome therapy

In LPS-induced ALI mice, TNF-α levels in BALF were significantly elevated across all exosome-treated groups compared to healthy controls [(132.7 ± 25.8) pg/ml], yet markedly reduced vs. the LPS model group [(499.4 ± 79.9) pg/ml]. Notably, intranasal medium-dose (Exo-VM: 305.8 ± 27.9 pg/ml), high-dose (Exo-NH: 355.7 ± 39.9 pg/ml), and nebulized medium-dose (Exo-AM: 313.9 ± 37.2 pg/ml) exosomes demonstrated significant TNF-α suppression (*P* < 0.05 vs. LPS model). Other dosages, including intravenous high-dose (Exo-VH: 327.3 ± 33.8 pg/ml), showed no statistical difference. Route-specific dose effects were observed: intravenous and nebulized deliveries exhibited no significant intra-dose variations (*P* > 0.05), whereas intranasal administration showed progressive reductions, with high- (Exo-NH) and medium-dose (Exo-NM) exosomes outperforming low-dose groups (*P* = 0.0010 and *P* = 0.0076, respectively), though high- and medium-dose efficacy did not differ (*P* = 0.2253). The balf TNF-α concentrations across treatment groups are further explored in Table 7.

**Table 7:** TNF-α concentrations across treatment groups.

Group	$\mathrm{TNF}\text{-}\alpha \;(\bar{X}\pm s)$	*P*-value
Control	132.7 ± 25.8	
LPS	499.4 ± 79.9*	
Exo-VL	450.7 ± 25.6*	0.9689
Exo-VM	415.4 ± 75.8*	0.4515
Exo-VH	424.7 ± 57.5*	0.6957
Exo-NL	480.3 ± 50.0*	>0.9999
Exo-NM	305.8 ± 27.9*^,^^#^	0.0005
Exo-NH	355.7 ± 39.9*^,^^#^	0.0174
Exo-AL	313.9 ± 37.2*^,^^#^	0.0022
Exo-AM	335.0 ± 33.3*^,^^#^	0.0042
Exo-AH	327.3 ± 33.8*^,^^#^	0.0025

Values expressed as mean ± SD $\left(\bar{X}\pm s\right)$ with sample size (*N* = 4) in parentheses <0.05 vs. control.

*Statistical significance compared to control group.

^#^ *P* < 0.05 vs. LPS model.

#### 3.3.2 IL-6 regulation in BALF following exosome therapy

Exosome treatment elevated IL-6 levels in BALF compared to healthy controls [(52.5 ± 10.8) pg/ml] across all experimental groups, yet only the intranasal medium-dose (Exo-NM: 134.1 ± 14.6 pg/ml) group demonstrated significant suppression vs. the LPS model group [(170.2 ± 2.8) pg/ml] (*P* = 0.0022). Other dosages, including intravenous and nebulized routes, showed no statistically meaningful reductions vs. LPS model (*P* > 0.05). Dose-dependent effects were absent within the same administration route: intravenous, intranasal, and nebulized deliveries exhibited no significant intra-dose variations (*P* > 0.05). The balf IL-6 concentrations across treatment groups are further explored in Table 8.

**Table 8:** IL-6 concentrations across treatment groups.

Group	$\mathrm{IL}\text{-}6\;(\bar{X}\pm s)$	*P*-value
Control	52.54 ± 10.8^#^	
LPS	170.2 ± 2.76*	
Exo-VL	149.4 ± 11.0*	0.2257
Exo-VM	162.0 ± 7.4*	0.9421
Exo-VH	152.5 ± 10.8*	0.2719
Exo-NL	152.5 ± 18.1*	0.2699
Exo-NM	134.1 ± 14.6*^,^^#^	0.0022
Exo-NH	149.6 ± 7.0*	0.2355
Exo-AL	157.3 ± 14.3*	0.5584
Exo-AM	149.2 ± 10.9*	0.1654
Exo-AH	155.1 ± 11.7*	0.4743

Values expressed as mean ± SD $\left(\bar{X}\pm s\right)$ with sample size (*N* = 4) in parentheses <0.05 vs. control.

*Statistical significance compared to control group.

^#^ *P* < 0.05 vs. LPS model.

#### 3.3.3 IL-1β modulation BALF following exosome therapy

Exosome treatment elevated IL-1β levels in BALF compared to healthy controls [(41.25 ± 8.7) pg/ml], with all experimental groups exceeding baseline levels. However, intravenous low-dose (Exo-VL: 149.4 ± 2.2 pg/ml), medium-dose (Exo-VM: 144.1 ± 4.5 pg/ml), high-dose (Exo-VH: 141.3 ± 8.7 pg/ml), intranasal medium-dose (Exo-NM: 147.6 ± 7.8 pg/ml), high-dose (Exo-NH: 141.1 ± 10.9 pg/ml), and aerosolized medium-dose (Exo-AM: 154.4 ± 10.7 pg/ml) exosomes demonstrated significant reductions vs. the LPS model group [(172.9 ± 10.3) pg/ml] (*P* < 0.05). Notably, no dose-dependent effects were observed within the same administration route, as intravenous, intranasal, and nebulized deliveries exhibited comparable IL-1β levels across low-, medium-, and high-dose groups (*P* > 0.05). The balf IL-1β concentrations across treatment groups are further explored in Table 9.

**Table 9:** IL-1β concentrations across treatment groups.

Group	$\mathrm{IL}\text{-}1\beta \;(\bar{X}\pm s)$	*P*-value
Control	41.3 ± 8.7	
LPS	172.9 ± 10.3*	
Exo-VL	149.4 ± 2.2*^,^^#^	0.0081
Exo-VM	144.1 ± 4.5*^,^^#^	0.0003
Exo-VH	141.3 ± 8.7*^,^^#^	<0.0001
Exo-NL	156.2 ± 6.8*	0.0999
Exo-NM	147.6 ± 7.8*^,^^#^	0.0018
Exo-NH	141.1 ± 10.9*^,^^#^	<0.0001
Exo-AL	165.1 ± 8.4*	0.7567
Exo-AM	154.4 ± 10.7*^,^^#^	0.0324
Exo-AH	158.9 ± 7.5*	0.2258

Values expressed as mean ± SD $\left(\bar{X}\pm s\right)$ with sample size (*N* = 4) in parentheses <0.05 vs. control.

*Statistical significance compared to control group.

^#^ *P* < 0.05 vs. LPS model.

#### 3.3.4 IL-10 upregulation in BALF following exosome therapy

Exosome treatment elevated IL-10 levels in BALF compared to healthy controls [(76.1 ± 26.5) pg/ml], with all experimental groups exceeding baseline levels. However, only intravenous high-dose exosomes (Exo-VH: 383.2 ± 94.1 pg/ml) demonstrated significant enhancement vs. the LPS model group [(227.5 ± 36.1) pg/ml] (*P* = 0.0254). Other dosages, including intranasal medium-dose (Exo-NM: 241.3 ± 32.7 pg/ml) and nebulized high-dose (Exo-AH: 267.5 ± 40.2 pg/ml), showed no statistical difference from the LPS model (*P* > 0.05). Dose-dependent effects were route-specific: intravenous delivery exhibited progressive IL-10 elevation, with high-dose (Exo-VH) outperforming low- (Exo-VL: 298.1 ± 45.6 pg/ml; *P* = 0.0122) and medium-dose (Exo-VM: 312.4 ± 38.9 pg/ml; *P* = 0.0108) groups, whereas low- and medium-dose efficacy did not differ (*P* = 0.9601). In contrast, intranasal and nebulized administrations showed no dose-dependent variations (*P* > 0.05). The balf IL-10 concentrations across treatment groups are further explored in Table 10.

**Table 10:** IL-10 concentrations across treatment groups.

Group	$\mathrm{IL}\text{-}10\;(\bar{X}\pm s)$	*P*-value
Control	76.10 ± 26.5	
LPS	227.5 ± 36.1*	
Exo-VL	254.0 ± 22.2*	0.9999
Exo-VM	244.3 ± 3.5*	>0.9999
Exo-VH	383.2 ± 94.1*^,^^#^	0.0254
Exo-NL	252.1 ± 7.1*	>0.9999
Exo-NM	265.8 ± 27.1*	0.9967
Exo-NH	244.8 ± 0.5*	>0.9999
Exo-AL	258.0 ± 54.0*	0.9991
Exo-AM	312.4 ± 98.2*	0.5477
Exo-AH	281.5 ± 68.3*	0.9407

Values expressed as mean ± SD $\left(\bar{X}\pm s\right)$ with sample size (*N* = 4) in parentheses <0.05 vs. control.

*Statistical significance compared to control group.

^#^ *P* < 0.05 vs. LPS model.

### 3.4 Therapeutic efficacy of exosome administration routes in LPS-induced ALI

**Figure 5:**
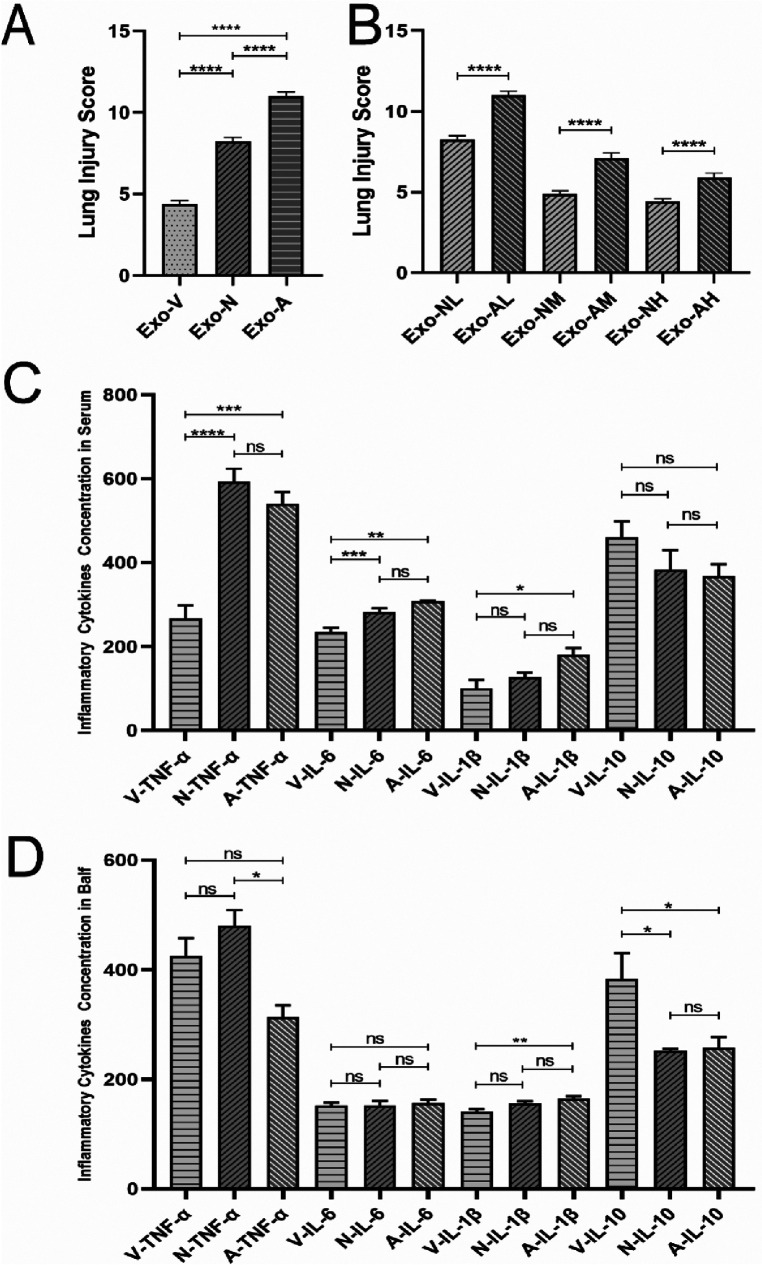
Comparative analysis of lung injury scores and cytokine levels in LPS-induced ALI mice at 48 hours post-treatment. **(A)** Comparison of lung injury scores among intravenous intranasal and nebulized groups. **(B)** Comparison of lung injury scores between intranasal and nebulizedgroups. **(C)** Serum cytokine concentration comparisons. **(D)** BALF cytokine concentration comparisons. *P < 0.05, **P < 0.005, *P* < 0.0001, ****P < 0.0001 vs. LPS model group.

At a standardized dose of 5 × 10⁸ particles per mouse, intravenous exosome delivery demonstrated superior therapeutic efficacy compared to intranasal and nebulized routes in mitigating LPS-induced ALI. Specifically, intravenous administration significantly reduced lung injury scores vs. intranasal delivery (*P* < 0.0001) and nebulized routes groups (*P* < 0.0001, Figure 5A), while intranasal delivery outperformed nebulized delivery (*P* < 0.0001, Figure 5B). Systemically, intravenous exosome delivery treatment markedly suppressed pro-inflammatory TNF-α and IL-6 levels in serum (*P* < 0.05) and elevated anti-inflammatory IL-10 concentrations in both serum and BALF (Figures 5C,D). In contrast, intranasal delivery and nebulized routes showed no significant differences in reducing pro-inflammatory cytokines (*P* > 0.05, Figures 5C,D), underscoring the critical role of route selection in optimizing exosome-mediated therapeutic outcomes.

## 4 Discussion

ALI and ARDS) are characterized by severe damage to alveolar, pulmonary endothelial, and epithelial cells, driven by mechanisms such as bacterial/viral invasion, immune hyperactivation, and mechanical ventilation-induced injury (27–29). Despite advances in clinical management—including antimicrobial therapies, corticosteroids, nutritional support, and mechanical ventilation—ARDS remains associated with high morbidity and mortality due to the lack of targeted therapies (30, 31). Emerging evidence highlights the pivotal role of cytokine storms in ARDS progression, where neutrophil and macrophage infiltration into alveolar spaces triggers inflammation, releasing pro-inflammatory cytokines such as TNF-α, IL-6, IL-1β, and IL-8. These cytokines exacerbate lung injury by activating pathways like NF-κB, amplifying inflammatory cascades, and disrupting alveolar-capillary barriers (32). For instance, TNF-α activates NF-κB signaling, driving inflammation and tissue damage (33), while IL-6 promotes neutrophil activation and aggregation (34), and IL-8 induces neutrophil migration, compromising endothelial and epithelial integrity (35). Thus, targeting immune balance to mitigate cytokine storms and protect alveolar/capillary barriers is critical for effective ARDS therapy.

Given the unresolved challenges in ALI/ARDS management, cell-based therapies have emerged as a promising avenue (16, 36). Mesenchymal stem cells (MSCs) have demonstrated therapeutic potential in preclinical and clinical studies by secreting anti-inflammatory cytokines, growth factors, and other bioactive molecules that modulate immune responses, reduce inflammation, and promote tissue repair (37–39). For example, MSC administration in murine ALI/ARDS models reduced lung injury, lowered cytokine levels, and improved survival rates (40). Our group previously reported that umbilical cord blood-derived stem cells repaired TLR4/MyD88/NF-κB signaling and stabilized immunity in an ARDS pediatric patient (41). Further mechanistic studies reveal that MSC-derived exosomes play a central role in these effects. Exosomes, as stable, non-immunogenic, and microvascular-safe intercellular communication vehicles, deliver miRNAs, proteins, and other bioactive components to regulate target cell gene expression and signaling pathways, exerting anti-inflammatory, anti-apoptotic, and tissue-repair effects (12, 13, 42–44). Notably, exosomal miRNAs inhibit pro-inflammatory cytokine production, reduce lung vascular permeability, and alleviate injury (45–48). Clinical trials also confirm that MSC-derived exosomes significantly reduce mortality in COVID-19-associated moderate-to-severe ARDS patients (20–22). However, pediatric applications of exosome therapy remain underexplored, and route-specific efficacy differences warrant further investigation.

This study investigated the therapeutic efficacy of exosome-based therapy in LPS-induced ALI/ARDS using a mouse model and compared the effects of different administration routes (intravenous, intranasal, and nebulized) across doses. Our results demonstrate that exosomes alleviate LPS-induced acute lung injury, as evidenced by reduced pro-inflammatory cytokine levels (TNF-α, IL-6, IL-1β) and elevated anti-inflammatory IL-10 in both serum and BALF, consistent with previous findings (49–51). Notably, intravenous delivery exhibited superior efficacy compared to intranasal and nebulized routes in suppressing TNF-α and IL-6 levels and ameliorating histopathological injury scores, while no significant differences were observed between intranasal and nebulized treatments. These findings highlight the critical role of administration route in optimizing exosome-mediated therapeutic outcomes, with intravenous delivery emerging as the most effective strategy for modulating inflammatory responses in ALI/ARDS.

Exosome biodistribution is influenced by multiple parameters, including stability, cellular origin, administration route, and composition (52, 53). Therefore, optimizing delivery routes and doses is critical for clinical translation. Exosomes can be administered via intravenous, subcutaneous, intranasal, intraperitoneal, or oral routes. While intravenous delivery has been extensively studied, intranasal and nebulized routes remain underexplored. Nebulization enables deep lung deposition, enhancing therapeutic targeting and anti-inflammatory effects (54, 55), whereas intranasal delivery, primarily used for neurological disorders (56), may also modulate respiratory immunity (57). In this study, intravenous exosome administration outperformed intranasal and nebulized routes in reducing histopathological injury scores and pro-inflammatory cytokine levels (TNF-α, IL-6, IL-1β) in LPS-induced ALI mice. This superiority may stem from intravenous mediated pulmonary targeting. Prior studies indicate that intravenous-injected exosomes preferentially accumulate in vascular-rich organs, including lungs, due to their interaction with the reticuloendothelial system (RES) (58, 59). For instance, intravenously administered MSC-derived exosomes rapidly localize to lungs within 3 h post-injection (59), though discrepancies exist (60, 61). Tolomeo et al. reported significant lung accumulation at 3 h post-intravenous injection in mice (62), while others observed predominant hepatic and renal sequestration in healthy animal. Disease context likely modulates biodistribution: MSC exosomes preferentially accumulate in injured tissues, such as kidneys in glycerol-induced nephropathy (63), or brains in hemorrhagic stroke models (64), suggesting injury-specific targeting. Similarly, in our LPS-ALI model, intravenous exosomes likely concentrated in damaged pulmonary endothelium, enhancing therapeutic efficacy. Notably, intravenous exosomes exhibit delayed systemic clearance compared to healthy controls, particularly under inflammatory conditions. While normal mice clear exosomes rapidly via macrophage/neutrophil phagocytosis, septic models show prolonged circulation (>80% exosomes retained at 1 h post-injection) (65, 66). Exosomes enter target cells via membrane fusion, receptor interactions, and endocytosis (67, 68), with pulmonary uptake primarily mediated by endothelial cells (69). Intravenous delivery enables rapid therapeutic effects by targeting endothelial cells in injured lung tissues. In contrast, no studies have reported similar mechanisms for intranasal or nebulized exosome delivery. A single study comparing intravenous and i.n. routes in opioid-addicted rats found no behavioral differences but higher bone marrow mesenchymal stem cell (BMMSC) engraftment in the intravenous group (70). Exosomes demonstrate delayed systemic clearance post-intravenous injection. While rapidly cleared by macrophages/neutrophils in healthy mice (45), ∼80% of exosomes persist in septic mice at 1 h post-injection (65). Whether nebulized or intranasal delivery induces similar delays remains unexplored. A prior comparison of nebulized and intravenous exosome distribution in bacterial pneumonia revealed global lung uptake for both routes, though nebulized achieved higher deposition (71), conflicting with our findings. Potential explanations include: (1) model heterogeneity (LPS-ALI vs. bacterial/viral pneumonia); (2) structural alterations during aerosolization or intranasal (mucociliary clearance); (3) exosome source/preparation variability. For instance, Antoin et al. observed superior survival with intravenous MSC microvesicles vs. intratracheal delivery in bacterial pneumonia (51), aligning with our results.

Notably, inflammatory cytokine reduction post-exosome therapy varies across timepoints. Studies report decreased TNF-α and IL-1β levels at 10 h, 24 h, 72 h, and even one week post-administration (1, 72–74). Our study focused on 48 h, confirming consistent anti-inflammatory effects, though optimal timing for ALI intervention requires multi-timepoint investigations.

To our knowledge, this is the first report evaluating i.n. exosome delivery for ALI treatment. However, limitations exist: (1) narrow model scope (pulmonary ALI in mice may not fully recapitulate human pathophysiology); (2) lack of longitudinal cytokine profiling and mechanistic exploration of MSC-EV therapeutic targets; and (3) limited sample size (though statistically sufficient, larger cohorts could capture subtle therapeutic nuances). Future studies should optimize delivery routes, elucidate molecular mechanisms, and validate findings in diverse ALI models to advance clinical translation.

## 5 Conclusion

This study shows that hUC-MSC-EVs reduce LPS-induced ALI in mice via pulmonary repair. Intravenous delivery outperformed intranasal and nebulized routes in mitigating lung injury at equivalent doses, underscoring the critical role of route selection.

## Data Availability

The original contributions presented in the study are included in the article/supplementary material, further inquiries can be directed to the corresponding author.
